# Bidirectional Influences of Cranberry on the Pharmacokinetics and Pharmacodynamics of Warfarin with Mechanism Elucidation

**DOI:** 10.3390/nu13093219

**Published:** 2021-09-16

**Authors:** Chung-Ping Yu, Meng-Syuan Yang, Pei-Wen Hsu, Shiuan-Pey Lin, Yu-Chi Hou

**Affiliations:** 1 School of Pharmacy, College of Pharmacy, China Medical University, Taichung 406040, Taiwan; yu1095813@gmail.com (C.-P.Y.); ymstiffany@gmail.com (M.-S.Y.); hsupw888@gmail.com (P.-W.H.); 2 Department of Pharmacy, China Medical University Hospital, Taichung 40447, Taiwan; 3 College of Medical and Health Science, Asia University, Taichung 41354, Taiwan

**Keywords:** cranberry, warfarin, supplement–drug interaction, pharmacokinetics/pharmacodynamics, breast cancer resistance protein, cytochrome P450

## Abstract

Cranberry is a dietary supplement popularly used for the prophylaxis of urinary tract infection. Interestingly, cranberry–warfarin interactions in clinical reports have shown bidirectional outcomes. (±) Warfarin, a widely prescribed anticoagulant, but with a narrow therapeutic index, contains equal amounts of S- and R-warfarin, of which S-warfarin is more active. The aim of this study was to investigate the effects of different ingestion times of cranberry on the pharmacokinetics and pharmacodynamics of warfarin. Rats were orally administered (±) warfarin (0.2 mg/kg) with and without cranberry (5.0 g/kg) at 0.5 h prior to the warfarin, and at 10 h after the warfarin. The plasma concentrations of S- and R-warfarin were determined by LC/MS. The results indicate that cranberry ingested at 0.5 h before (±) warfarin significantly decreased the systemic exposures of S-warfarin and R-warfarin. Conversely, when cranberry was ingested at 10 h after (±) warfarin, the elimination of S-warfarin was significantly inhibited, and the anticoagulation effect of (±) warfarin was significantly enhanced. The results of the mechanism studies indicate that cranberry activated the breast cancer resistance protein (BCRP), which mediated the efflux transports of S-warfarin and R-warfarin. Moreover, the metabolites of cranberry inhibited cytochrome P450 (CYP) 2C9, the main metabolizing enzyme for S-warfarin. In conclusion, cranberry affected the pharmacokinetics of (±) warfarin in a bidirectional manner by activating the BCRP by CJ during absorption and inhibiting the BCRP and CYP2C9 by CMs during elimination, depending on the ingestion time of CJ. The combined use of cranberry with warfarin should be avoided.

## 1. Introduction

Cranberry is the fruit of *Vaccinium microcarpum*, *V. oxycoccos*, or *V. erythrocarpum* (Ericaceae). Nowadays, cranberry-based supplements have been popularly used for the prophylaxis of urinary tract infections, especially for the elderly [1]. According to a market report, cranberry is the third top-selling supplement [2]. Cranberry contains a variety of polyphenols, including proanthocyanidins, anthocyanins, flavonoids, and phenolic acids, which exhibit numerous beneficial effects, such as the prevention of microbial infections, cardiovascular diseases, and cancers, and the improvement of lipid profiles and digestive disorders [1].

(±) Warfarin, a vitamin K antagonist, is a widely prescribed oral anticoagulant for the prevention and treatment of thromboembolic disorders and recurrent transient ischemic attacks, as well as for reducing the risk of recurrent myocardial infarction [3]. Being acidic (pKa = 4.94), (±) warfarin exists as anions in the body. Recently, the transport of S- and R-warfarin was proven through the mediation of the breast cancer resistance protein (BCRP) [4], which was one of the ATP-binding cassette transporters with a diverse range of substrates, including those both positively and negatively charged, as well as large amphiphilic molecules [5]. In clinical practice, warfarin is administered as a racemic mixture, composed of S- and R-warfarin (1:1), of which S-warfarin was 2 to 5 times more potent than R-warfarin in terms of anticoagulant effect [6]. In a pharmacokinetic context, S-warfarin was mainly metabolized by cytochrome P450 (CYP) 2C9, whereas R-warfarin was metabolized by CYP1A2 and CYP3A4 [7].

In recent decades, the concurrent use of dietary supplements with prescription drugs is prevalent in chronic patients, and the risks of dietary supplement–drug interactions have increased [8,9]. Among clinical cranberry–drug interactions, warfarin was the most discussed medicine due to the reports of bidirectional critical outcomes, including bleeding and a diminished anticoagulation effect [10,11,12]. In a clinical setting, the safety and efficacy of warfarin therapy depend on the maintenance of the international normalized ratio (INR) within the target ranges (2.0~3.0 or 2.5~3.5) for the indications of different disease states [13]. Therefore, routine INR monitoring is essential and recommended for all patients receiving warfarin therapy [13].

Cranberry polyphenols and the relevant metabolites have been reported as substrates and/or inhibitors of the BCRP [14,15,16,17]. In addition, cranberry polyphenols have shown inhibitions on the activities of CYP2C9 and CYP3A [18,19,20,21]. We hypothesized that cranberry ingestion might affect the BCRP-mediated transport and CYP-catalyzed metabolism of S- and R-warfarin. Until now, the controversial outcomes of cranberry–warfarin interaction remained unexplainable by any known mechanisms [22,23]. Therefore, this study was an investigation into the influences of different ingestion times of cranberry on the pharmacokinetics and pharmacodynamics of (±) warfarin. Furthermore, the probable mechanisms related to the BCRP and CYPs were explored.

## 2. Materials and Methods

### 2.1. Chemicals and Reagents

(±) Warfarin sodium, caffeic acid, rutin, myricetin, quercetin, kaempferol, protocatechuic acid, isorhamnetin, 6,7-dihydroxycoumarinand formic acid were obtained from Sigma-Aldrich Chemical Co. (St. Louis, MO, USA). S-warfarin and R-warfarin were purchased from Toronto Research Chemicals, Inc. (North York, ON, Canada). Scopolatin was obtained from Tokyo Chemical Industry Co. (Tokyo, Japan). Cyanidin chloride was purchased from ChromaDex, Inc. (Irvine, CA, USA). Acetonitrile and LC/MS grade methanol were obtained from J.T. Baker Inc. (Center Valley, PA, USA). Mitoxantrone (MXR) and Ko143 were obtained from Enzo Life Sciences, Inc. (New York, NY, USA). Dulbecco’s modified Eagle medium (DMEM), fetal bovine serum (FBS), and trypsin/EDTA were obtained from Invitrogen (Carlsbad, CA, USA). Milli-Q plus water (Billerica, MA, USA) was used for all preparations.

### 2.2. Characterization of Cranberry Juice (CJ)

CJ was prepared from Ben Lear cranberries imported from Canada. A total of 1.5 kg of cranberries was blended with 1.5 L of water using a Thermomix TM31 (Vorwerk & Co. KG, Wuppertal, Germany). Then, sufficient water was added to make 3 L to afford 0.5 g/mL of CJ. The CJ was mixed with methanol (*v*/*v*, 3:7) and centrifuged. The supernatant (200 μL) was mixed with 200 μL of methanolic solution of internal standard (6,7-dimethoxycoumarin, 0.01 μg/mL), and 5 μL was subject to LC/MS/MS analysis, which was modified from a previous study [24]. Briefly, the mobile phase consisted of 0.01% formic acid (A) and acetonitrile containing 0.01% formic acid (B), and a gradient elution was programmed as follows: A/B: 80/20 (0–1.0 min), 20/80 (1.1–4 min), and 80/20 (4.1–8.0 min). The flow rate was 0.2 mL/min. Nitrogen was used as a sheath gas at 40 arbitrary units and as an auxiliary gas at 10 arbitrary units. The collision energy was set at 38 V, the spray voltage at 3000 V, the capillary temperature at 270 °C, the vaporizer temperature at 300 °C, and the tube lens offset at 156 V. The following mass transitions were used for selected reaction monitoring (SRM) analysis: protocatechuic acid (153/108), scopoletin (191/176), resveratrol (227/185), rutin (609/301), myricetin (317/151), quercetin (301/151), kaempferol (285/151), isorhamnetin (315/151). The ESI-MS spectra were recorded in negative ion mode, whereas cyanidin (287/213) and the internal standard 6,7-dimethoxycouumarin (207/151) were recorded in positive ion mode.

### 2.3. Animals

Male Sprague–Dawley rats, weighing 300–450 g, were purchased from BioLASCO Taiwan Co., Ltd. (Taipei, Taiwan). The animal study adhered to *The Guidebook for the Care and Use of Laboratory Animals* published by the Chinese Society for the Laboratory Animal Science, Taiwan, and the experimental protocol had been approved by the Institutional Animal Care and Use Committee of China Medical University (Taichung, Taiwan).

### 2.4. Administrations of CJ and Warfarin, and Blood Collection

Before the experiment, the rats were fasted overnight but drinking water was allowed ad libitum. Isoflurane (3%) was used for anesthesia prior to each blood sampling. Food was supplied at 3 h after warfarin dosing.

In the pharmacokinetic interaction study, the rats were randomly divided into three groups (n = 7 in each group). The first group of rats was orally administered (±) warfarin (0.2 mg/kg) alone. The second and third groups of rats were administered CJ (5 g/kg) at 0.5 h prior to (±) warfarin and at 10 h after (±) warfarin dosing, respectively. The study was performed in a parallel design. Blood samples were drawn at 0.5, 1.0, 2.0, 6.0, 10, 24, 48, 72, and 96 h after (±) warfarin dosing and collected in a Vacutainer K2 EDTA tube (BD, New Jersey, USA). The blood samples were centrifuged to obtain plasma.

In the pharmacodynamic study, the rats were randomly divided into four groups (n = 8 in each group). In the first experiment, two groups of rats were orally administered (±) warfarin (0.2 mg/kg) alone and with CJ (5 g/kg) given at 0.5 h prior to (±) warfarin dosing, and blood samples were drawn at 6, 10 and 24 h after (±) warfarin dosing. In the second experiment, two groups of rats were orally administered (±) warfarin (0.2 mg/kg) with and without CJ (5 g/kg), given at 10 h after (±) warfarin dosing, and blood samples were drawn at 10, 24, and 48 h after (±) warfarin dosing.

### 2.5. Quantitation of S- and R-Warfarin in Plasma

The plasma concentrations of S- and R-warfarin were simultaneously quantitated by the LC/MS method, modified from our previous study [4]. Briefly, 100 µL of plasma was mixed with 50 µL of 0.5N formic acid and then partitioned with 150 µL of ethyl acetate containing 10 µg/mL of caffeic acid as an internal standard.

### 2.6. Measurement of INR in Rats

Ten µL of blood was collected and the INR was measured using CoaguChek^®^ XS System (Roche Diagnostics GmbH, Mannheim, Germany). All procedures followed the manufacturer’s protocol.

### 2.7. Cell Lines and Culture Conditions

Human BCRP-transfected Madin–Darby canine kidney II cells (MDCKII-BCRP) were kindly provided by Prof. Dr. Piet Borst (Netherlands Cancer Institute, Amsterdam, The Netherlands). Cells were grown in DMEM supplemented with 10% FBS, 100 units/mL of penicillin, 100 μg/mL of streptomycin, and 292 μg/mL of glutamine at 37 °C in a humidified incubator containing 5% CO_2_. The medium was changed every other day and the cells were subcultured when 80% to 90% confluency was reached.

### 2.8. Preparation of Cranberry Metabolites (CMs)

In order to mimic the real molecules interacting with CYPs and the BCRP in the enterocytes, hepatocytes, and nephrocytes after CJ intake, CMs were prepared from the rats given CJ. Briefly, rats were orally administered with CJ (5 g/kg), and the blood was collected at 30 min after CJ intake. After coagulation and centrifugation, the serum was vortexed with 4-fold methanol and furthermore centrifuged. The supernatant was concentrated in a rotatory evaporator under vacuum to dryness. In addition, blank serum was collected from the rats given the same volume of water as CJ and processed similarly to prepare a blank serum control for comparison with the CMs.

### 2.9. Effects of CJ and CMs on BCRP-Mediated Efflux

MDCKII-BCRP cells were used to evaluate the effects of CJ and CMs on BCRP-mediated efflux of mitoxantrone (MXR), a fluorescent typical substrate of the BCRP, and Ko143 was used as a positive control for the BCRP inhibitor. Furthermore, all procedures were conducted according to our previous study [25].

### 2.10. Effect of CMs on the Activities of CYP1A2, CYP2C9, and CYP3A4

Vivid^®^ CYP450 screening kits were used to evaluate the effects of CMs on the activities of CYP1A2, CYP2C9, and CYP3A4, individually [26,27], and all the procedures were performed based on the standard protocols. In these assays, α-naphthoflavone (ANF, 3 μM), sulfaphenazole (SFZ, 10 μM), and ketoconazole (Keto, 10 μM) were used as positive inhibitor controls for CYP1A2, CYP2C9, and CYP3A4, respectively.

### 2.11. Data Analysis

The pharmacokinetic parameters were calculated by Phoenix WinNonlin^®^ version 8.1 (Pharsight Corporation, St. Louis, MO, USA). The unpaired Student’s *t*-test and one-way ANOVA were used for statistical comparisons, taking *p* < 0.05 as significant.

## 3. Results

### 3.1. Characterization of CJ

**Figure 1:**
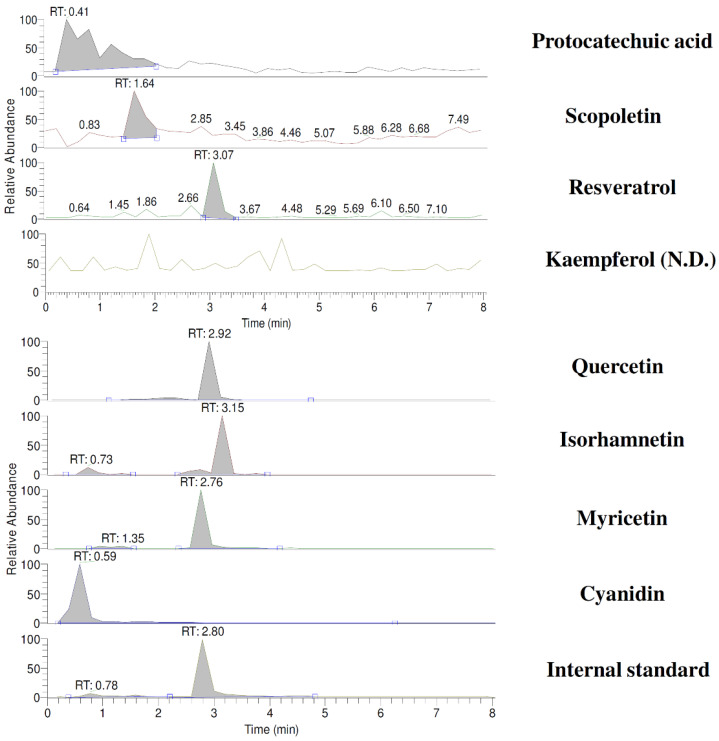
LC/MS/MS chromatograms of cranberry juice. SRM: protocatechuic acid (*m*/*z* 153/108); scopoletin (*m*/*z* 191/176); resveratrol (*m*/*z* 227/185); kaempferol (*m*/*z* 285/151); quercetin (*m*/*z* 301/151); isorhamnetin (*m*/*z* 315/151); myricetin (*m*/*z* 317/151); cyanidin (*m*/*z* 287/213); internal standard, 6,7-DMC (*m*/*z* 207/151).

The known constituents of CJ that were well resolved in the LC/MS/MS chromatogram within 8 min are shown in Figure 1. The quantitation results indicate that a dose of 5.0 g/kg of CJ contained 4.4, 0.7, 6.1, 856.5, 6.2, and 0.5 μg/kg of quercetin, isorhamnetin, myricetin, cyanidin, protocatechuic acid, and scopoletin, respectively, indicating that cyanidin was the major constituent in CJ.

### 3.2. Effect of CJ on the Pharmacokinetics of S- and R-Warfarin in Rats

**Figure 2:**
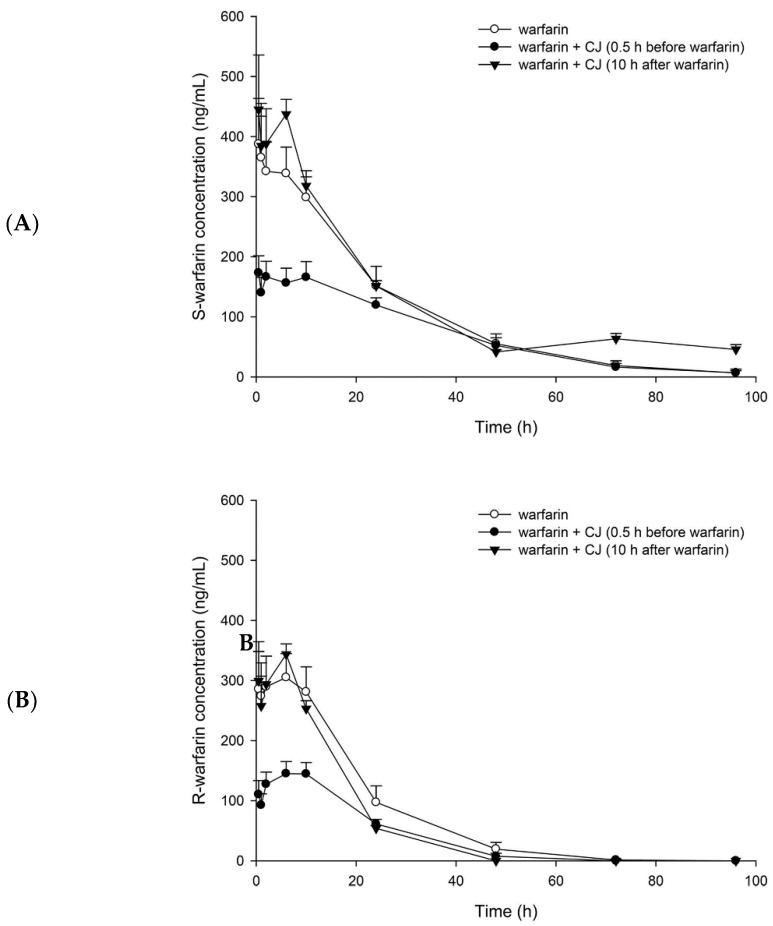
Mean (±S.E.) plasma concentration–time profiles of S-warfarin (**A**) and R-warfarin (**B**), after oral administration of (±) warfarin (0.2 mg/kg) alone and coadministration with cranberry juice (CJ, 5.0 g/kg) at 0.5 h before and 10 h after (±) warfarin in rats.

Figure 2 depicts the plasma concentration–time profiles of S- and R-warfarin in rats after oral administration of (±) warfarin with and without CJ ingestion at 0.5 h prior to (±) warfarin, and at 10 h after (±) warfarin dosing. The pharmacokinetic parameters are listed in Table 1. The results show that CJ ingestion at 0.5 h before (±) warfarin significantly decreased the C_max_ and AUC_0-t_ of S-warfarin by 48% and 34%, and reduced the C_max_ and AUC_0–t_ of R-warfarin by 51% and 52%, respectively. In addition, the AUC_0–10_ of S- and R-warfarin were decreased by 52% and 54%, respectively. In another study, when CJ was given at 10 h after (±) warfarin, the C_max_ and AUC_0–t_ of S- and R-warfarin were not affected. Nevertheless, the t_1/2_ and AUC_48–96_ of S-warfarin were significantly increased, by 267% and 126%, respectively.

### 3.3. Influence of CJ on the Anticoagulation Effect of Warfarin in Rats

**Figure 3:**
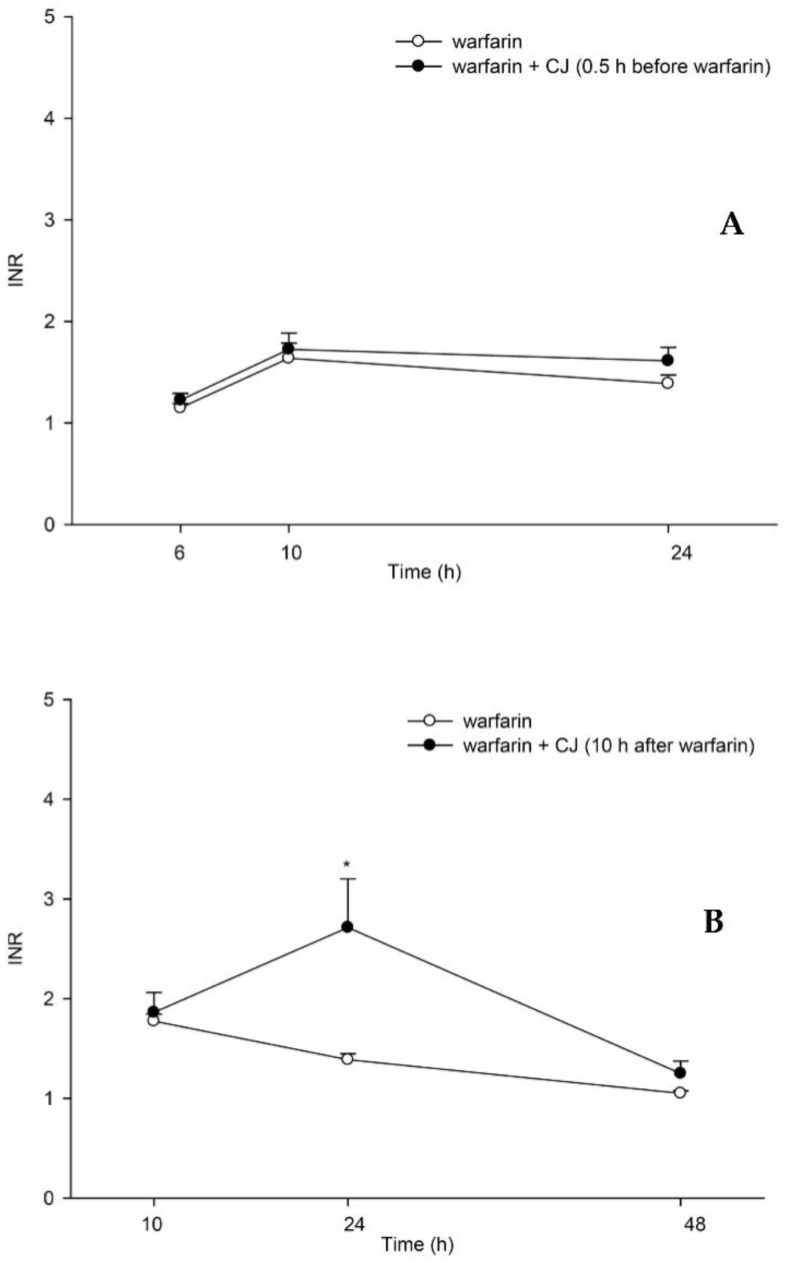
Mean (±S.E.) INR–time profiles after oral administration of (±) warfarin (0.2 mg/kg) alone and coadministration with cranberry juice (CJ, 5.0 g/kg) at 0.5 h before (±) warfarin (**A**) and 10 h after (±) warfarin (**B**) in rats. * *p* < 0.05.

The mean INR-time profiles after oral administration of (±) warfarin with and without CJ ingestion at 0.5 h before and 10 h after warfarin dosing are shown in Figure 3. The results indicate that when CJ was given 10 h later, the INR value at 24 h increased to 2.7 ± 0.5, which was significantly higher than that of the control rats (1.4 ± 0.1), by 93%. In contrast, when CJ was given at 0.5 h prior to warfarin, the INR value at 24 h was 1.6 ± 0.1, which was not different from the control rats.

### 3.4. Effects of CJ and CMs on the Activity of the BCRP

**Figure 4:**
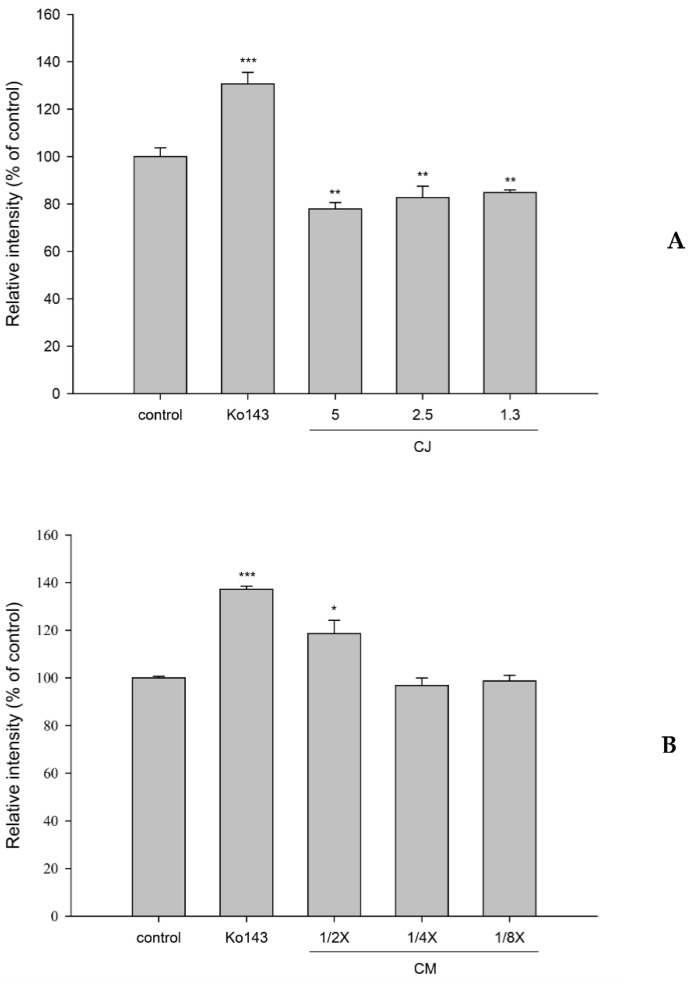
Effects of cranberry juice (CJ, mg/mL) (**A**) and serum metabolites of cranberry (CMs, at one half, one quarter and one eighth of the serum concentrations) (**B**) on the intracellular accumulation of MXR in MDCKII-BCRP cells. * *p* < 0.05, ** *p* < 0.01, and *** *p* < 0.001. Ko143: a positive control for the BCRP inhibitor.

The effects of CJ and CMs on the intracellular accumulation of MXR in MDCKII-BCRP cells are shown in Figure 4. The results show that 5.0, 2.5, and 1.3 mg/mL of CJ significantly decreased the intracellular accumulation of MXR by 22%, 17%, and 15%, while Ko143 increased that by 31%. Conversely, CMs at one half of the serum concentration significantly increased the intracellular accumulation of MXR by 19%. Ko143, spiked in blank serum, significantly increased it by 37%.

### 3.5. Effect of CMs on the Activities of CYP1A2, CYP2C9, and CYP3A4

**Figure 5:**
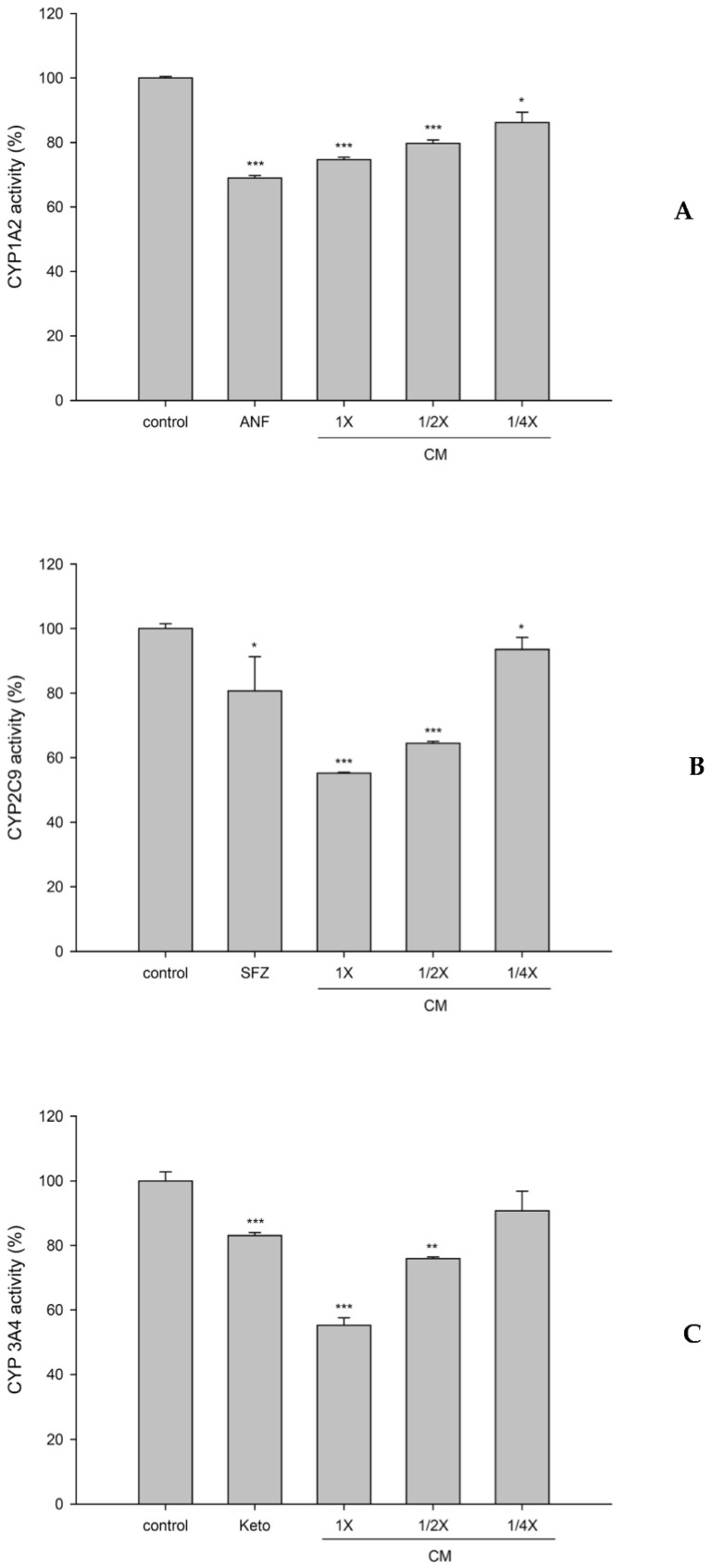
Effects of cranberry serum metabolites (CM; full, one half, and one quarter of the serum concentrations) on the activities of CYP1A2 (**A**), CYP2C9 (**B**), and CYP3A4 (**C**). * *p* < 0.05, ** *p* < 0.01, and *** *p* < 0.001. ANF (α-naphthoflavone): a positive control for the CYP1A2 inhibitor. SFZ (sulfaphenazole): a positive control for the CYP2C9 inhibitor. Keto (ketoconazole): a positive control for the CYP3A4 inhibitor.

Figure 5 shows the effects of CMs on the activities of CYP1A2, CYP2C9, and CYP3A4. The results revealed that CMs at full, one half, and one quarter of the serum concentrations significantly decreased the activity of CYP1A2 by 25%, 20%, and 14%, respectively, when compared to those of corresponding concentrations of blank serum. ANF, a positive control for the CYP1A2 inhibitor in blank serum, significantly decreased it by 31%.

In addition, CMs at full, one half, and one quarter of the serum concentrations significantly decreased the activity of CYP2C9 by 45%, 36%, and 7%, respectively. SFZ, a positive control for the CYP2C9 inhibitor in blank serum, significantly decreased it by 19%.

On other hand, CMs at full and one half of the serum concentrations significantly decreased the activity of CYP3A4 by 45% and 24%, respectively. Keto, a positive control for the CYP3A4 inhibitor in blank serum, significantly decreased it by 17%.

## 4. Discussions

The simultaneous determination of S- and R-warfarin in plasma was performed by employing an LC/MS method [4]. The discrepant profiles between S- and R-warfarin shown in Figure 2 reveal the stereoselective pharmacokinetics of warfarin, which is in good agreement with a previous study [28]. The profiles of S-warfarin, the more active form, were higher than that of R-warfarin following each treatment, implying that S-warfarin was absorbed more, metabolized less, or excreted slower than R-warfarin.

The results of the pharmacokinetic study showing that CJ ingestion prior to warfarin decreased the C_max_ and AUC_0-t_ of both S- and R-warfarin indicate that CJ reduced the bioavailabilities of S- and R-warfarin, which is in agreement with the decreased efficacy of warfarin alerted by a few clinical reports [22,29]. Noticeably, the AUC_0-t_ of S-warfarin was decreased by 34%, which was less than that of R-warfarin (by 52%), indicating that CJ reduced the bioavailability of S-warfarin to a lesser extent. Otherwise, the pharmacokinetic interaction of CJ with warfarin would result in a higher risk of deteriorating the efficacy of warfarin.

In contrast, another treatment giving CJ at 10 h after warfarin was intentionally designed to avoid the influence of CJ on the absorption of warfarin. The results show that the profile of S-warfarin was apparently higher than control in the terminal phase, whereas the profile of R-warfarin was not altered, indicating that CJ inhibited the elimination of S-warfarin, but not R-warfarin. This stereoselective phenomenon is similar to the interactions of warfarin with metronidazole [30] and cimetidine [31].The comparison of pharmacokinetic parameters showing that CJ increased the half-life and the terminal exposure (AUC_48–96_) of S-warfarin further confirmed that the elimination of S-warfarin was inhibited. The decreased elimination of S-warfarin caused by CJ might lead to an increased INR or even bleeding, which is consistent with the alerts of several clinical reports [10,12,32,33].

Based on the pharmacokinetic finding, a pharmacodynamic assessment of warfarin was subsequently performed through the determination of the INR in rats. The results show that INR was significantly enhanced at 24 h post-warfarin dosing when CJ was given at 10 h later, which could echo previous clinical alerts of bleeding or an enhanced INR due to cranberry–warfarin interaction [10,12,32,33]. Taken together with the pharmacokinetic data, we can conclude that the delayed ingestion of CJ enhancing the INR was through inhibiting the elimination of S-warfarin. In contrast, when CJ was given prior to warfarin, the INR was not different from the control.

How did the delayed ingestion of CJ inhibit the elimination of S-warfarin? The pharmacokinetics of polyphenols showed rapid and extensive metabolism to form conjugated metabolites, such as glucuronides and sulfates, which were acids existing as anions in the body, and their excretions were mediated by transporters, such as the BCRP and MRPs [34]. These conjugated metabolites were probable inhibitors of the BCRP. Therefore, the CMs were prepared for mimicking the real molecules interacting with the BCRP and metabolizing enzymes. A transport assay indicated that CMs inhibited the transport of the BCRP, which could, in part, account for the decreased excretion of S-warfarin. The inhibition of CMs on the BCRP is similar to the modulation of resveratrol metabolites reported recently [27]. We speculate that the delayed ingestion of CJ might increase the systemic exposure of various substrate drugs of the BCRP.

How did concomitant ingestion of CJ reduce the absorption of S- and R-warfarin? A transport assay indicated that CJ activated the efflux of the BCRP, which could, in part, account for the reduced absorption of both S- and R-warfarin in the intestine. We speculate that concomitant ingestion of CJ might decrease the absorption of various substrate drugs of the BCRP.

Regarding the modulations of CJ on metabolizing enzymes, this was the first study employing CMs, the virtual molecules interacting with CYPs, for evaluation. The results showing that CMs inhibited the activity of CYP2C9, the main metabolizing enzyme of S-warfarin, could explain, in part, the decreased elimination of S-warfarin following delayed ingestion of CJ. However, although the activities of CYP1A2 and CYP3A4 were likewise inhibited by CMs, no significant change to the pharmacokinetics of R-warfarin was observed. Based on the present ex vivo findings, we speculate that other than warfarin, the pharmacokinetics, efficacy, and safety of other substrate drugs of CYP1A2, CYP2C9, or CYP3A4 are likely altered by cranberry.

This study was the first one to disclose that the timing of cranberry ingestion is a key factor leading to opposite outcomes of cranberry–warfarin interaction. The results could explain the controversial findings of cranberry–warfarin interactions in clinical reports [10,11,12]. We suggest that the concurrent use of cranberry with warfarin should be avoided in order to ensure the safety and efficacy of warfarin.

## 5. Conclusions

Concomitant ingestion of CJ reduced the systemic exposure of S- and R-warfarin. Conversely, delayed ingestion of CJ prolonged the half-life of S-warfarin and enhanced the anticoagulation effect of warfarin. The mechanisms were activating the BCRP by CJ during absorption and inhibiting the BCRP and CYP2C9 by CMs during elimination.

## Figures and Tables

**Table 1:** Pharmacokinetic parameters of S- and R-warfarin after oral administrations of (±) warfarin (0.2 mg/kg) with and without cranberry juice (CJ, 5.0 g/kg) at 0.5 h before and 10 h after warfarin to 7 rats in each group.

Compound	Parameters	Warfarin Alone	Warfarin + CJ (0.5 h before Warfarin)	Warfarin + CJ (10 h after Warfarin)
S-warfarin	C_max_	418.2 ± 69.4 ^a^	216.3 ± 18.1 ^b^	542.0 ± 59.4 ^a^
			(−48%)	
	AUC_0–t_	9459.1 ± 1659.6 ^a^	6286.7 ± 456.3 ^b^	11,555.0 ± 613.6 ^a^
			(−34%)	
	AUC_0–10_	3267.7 ± 423.2 ^a^	1561.8 ± 217.3 ^b^	3846.6 ± 260.8 ^a^
			(−52%)	
	AUC_48–96_	1126.6 ± 434.5 ^a^	1041.3 ± 296.5 ^a^	2547.9 ± 339.5 ^b^
				(+126%)
	t_1/2_	15.5 ± 2.4 ^a^	19.1 ± 3.2 ^a^	56.9 ± 8.0 ^b^
				(+267%)
R-warfarin	C_max_	341.1 ± 56.6 ^a^	168.6 ± 17.2 ^b^	402.8 ± 33.6 ^a^
			(−51%)	
	AUC_0–t_	6209.2 ± 1253.4 ^a^	3007.6 ± 230.0 ^b^	4739.3 ± 255.0 ^ab^
			(−52%)	
	AUC_0–10_	2848.9 ± 395.0 ^a^	1310.8 ± 169.6 ^b^	2943.7 ± 185.7 ^a^
			(−54%)	
	AUC_48–96_	222.0 ± 125.3	90.4 ± 58.2	0.0
	t_1/2_	10.0 ± 1.3 ^ab^	12.6 ± 2.2 ^a^	6.7 ± 0.3 ^b^

Data expressed as mean ± S.E. Means in a row without a common superscript differ. (*p* < 0.05). A mean with a symbol of “^a^” is significantly different from a mean with a symbol of “^b^”. C_max_ (ng/mL): maximum concentration. AUC_0–t_ (h⋅ng/mL): area under concentration–time curve to the last time. AUC_0–10_ (h⋅ng/mL): area under concentration–time curve from 0 to 10 h. AUC_48–96_ (h⋅ng/mL): area under concentration–time curve from 48 to 96 h. t_1/2_ (h): elimination half-life.

## Data Availability

The data that support the findings of this study are not available.
